# Reconstruction of Gingival Mask around CAD-CAM Bar and Ball Attachment in Implant-Retained Overdenture

**DOI:** 10.1155/2024/4166767

**Published:** 2024-01-08

**Authors:** Samira Naybandi Atashi, Somayeh Zeighami

**Affiliations:** Department of Prosthodontics, School of Dentistry, Tehran University of Medical Sciences, Tehran Po. Code: 14399-55991, Iran

## Abstract

Gingival mask is a copy of the peri-implant tissue, which plays an important role in the fabrication of an optimal restoration. Losing the gingival mask is a clinical problem that complicates the process of restoration fabrication. Herein, a simple precise technique is described step by step to solve this problem in the patient with CAD/CAM milled bar and ball attachment treatment plan for a maxillary and a mandibular implant-supported overdenture, without the need to repeat the entire clinical and laboratory procedures.

## 1. Introduction

Using implants for edentulous patients has given hope to obtaining a prosthesis that is adequately retained, stable, and comfortable as well [1]. Broadly, overdenture attachment systems are divided into five main categories: ball, locator, bar, magnet type, and telescopic attachments. The selection of attachment type depends on various factors such as interarch space, angulation between the implants, and patient's economic condition. The highest required space (between the tooth incisal edge to the mucosa) is related to the bar attachment (13–14 mm), and the lowest is related to the magnet attachment (8.5 mm). The angle between the implants can be corrected better in the bar or locator attachment than in the telescopic or ball attachment [2].

Implant overdenture bars are traditionally fabricated using the lost-wax technique and conventional casting method, which is time-consuming and labour-intensive. Alternatively, the overdenture bar framework can be fabricated using the CAD/CAM method. Occasionally, the fabrication of one-piece cast implant frameworks may encounter issues like potential misfits and porosities [3]. CAD/CAM has proved to have higher precision and accuracy [4]. The improved accuracy can be attributed to multiple factors. Firstly, it benefits from the use of fewer fabrication steps, which have their own inherent margin of inaccuracy. CAD/CAM fabrication eliminates impression, cast pouring, investing, and alloy casting stages. Additionally, the accuracy of scanner and milling machine used in this process may contribute to the overall precision, surpassing that of traditional laboratory techniques [5]. The accuracy and fit of these CAD/CAM frameworks have been shown to be more accurate than one-piece cast framework by a number of studies [5–8]. CAD/CAM implant frameworks offer potential cost savings compared to one-piece cast frameworks due to the use of titanium alloy instead of noble alloy. They are also lighter in weight, and the locator or ball attachments are securely screwed into a milled screw base, resulting in consistent insertion axis and reduced wear. Additionally, locators or ball attachments can be replaced individually without replacing the entire framework. In contrast, the conventional casting method may introduce errors during attachment placement. Redoing the CAD/CAM framework is easier as the same design file can be used without the need for a new impression [9]. In 2010, Bueno et al. stated that the implant-supported milled bar overdenture is a very interesting option in the treatment of patients with moderate-to-severe reabsorbed maxilla problems. It offers both the advantages of removable prostheses and the stability and retention of a fixed prosthesis [10].

In order to achieve better conformity of the prosthetic restoration made to the patient's gum and esthetics, the patient's gingival form should be reconstructed; to do this, soft material is used to let the dentist have the patient's gingival form in the laboratory [11].

Gingival mask is a highly precise copy of the peri-implant gingival tissue, which aids in more accurate designing of prosthetic restoration, superior oral hygiene, and improvement of periodontal condition. Also, gingival mask allows the observation of precise seating of the suprastructure on implant analog and plays a fundamental role in the fabrication of a suprastructure with an ideal fit [12, 13]. Several materials are used as gingival mask, such as polyether impression material and silicone material [14].

Two methods are commonly used for the fabrication of gingival mask:

- Direct method: an implant impression is made, the gingival mask is placed at the respective site, and the gypsum cast is poured [12, 13]
- Indirect method: an implant impression is made, the gypsum cast is poured, and the gingival mask space is created by trimming the cast. A silicone index is tightened on the cast by a screw-retained abutment, and gingival mask is injected into the site through the silicone index holes (according to manufacturer instructions)

In a number of articles, different methods have been used to make a new gingival mask on the patient's previous cast after forming the emergence profile with a temporary restoration in patients with a fixed implant restoration treatment plan [15, 16].

In 2015, Esguerra [15] suggested the pickup technique of provisional restorations for the fabrication of a special gingival mask in full-arch fixed implant restorations. In this method, the impression copings are connected to each other intraorally by using acrylic resin, and then, analogs are tightened over them. Type IV dental stone is poured into the base former, and analogs are mounted in dental stone by half of their length. The provisional restoration is tightened intraorally and a pickup impression is made and transferred to the cast, and the space between the cast and impression is filled with gingival mask [15].

In 2018, Tse and Marchack [16] used the vacuum sheet and gingival mask injection technique to transfer the emergence profile form from a provisional restoration to final restoration. In this method, an impression is made intraorally, and the master cast is poured. Next, a provisional restoration is fabricated for the patient, and the emergence profile is corrected by composite resin. A pickup impression is made from the provisional restoration intraorally, the cast is poured, and the vacuum sheet is formed over it. The provisional restoration along with the vacuum sheet is transferred to the master cast. Some holes are created in the vacuum sheet for the injection of gingival mask, and the gingival mask is subsequently injected at the site [16].

Fabrication of fixed or removable implant-supported full-arch restorations is a complex procedure that requires several clinical and laboratory sessions. In this process, the dental clinician and technician often need to frequently remove the gingival mask from the cast for precise fabrication of suprastructure and observation of its perfect fit over the cast, which increases the risk of losing the gingival mask. Currently, no alternative is available for this method. Losing a gingival mask would necessitate repeating the entire process. In our research, no study was found to solve this problem without repeating the entire process. Herein, a simple precise technique is described step by step to solve the problem of losing the gingival mask in the patient with CAD/CAM milled bar and ball attachment treatment plan for a maxillary and a mandibular overdenture, without the need to repeat the entire clinical and laboratory procedures.

## 2. Case Report

The definition of abbreviations and techniques is given in Table 1. The patient's informed consent was obtained for the publication of this case report. The patient was a 58-year-old man who had been referred to the Faculty of Dentistry at Tehran University of Medical Sciences with a complaint of toothlessness. He had 4 implants in each upper and lower jaw in the area of teeth 12, 14, 22, 24, 32, 34, 42, and 44. The distance between the implants was about 1 cm, except for the two anterior maxillary implants, which were 2 cm apart. The implant-supported overdenture treatment plan was considered for the patient. After the initial arrangement of the teeth, due to the large interarch space and inappropriate angulation between the implants (especially the two anterior maxillary implants), the milling bar and ball attachment system were selected.

At the stage of trying the milling bar and ball resin patterns in the mouth, it was found that the gingival masks of both casts were lost in the transfer of the casts from the laboratory to the clinic. To solve this problem without adding the patient's treatment sessions, the following steps were performed in order.

**Figure 1:**
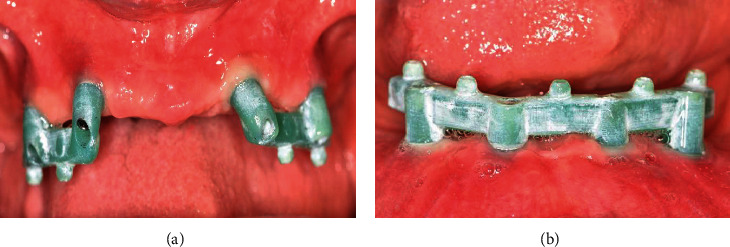
Resin pattern. (a) Maxillary arch. (b) Mandibular arch.

**Figure 2:**
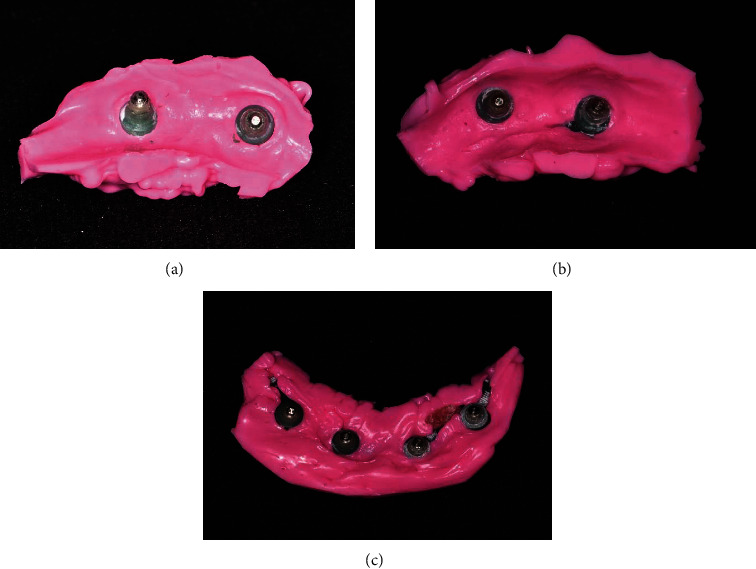
Impression with resin patterns. (a, b) Maxillary impression. (c) Mandibular impression.

**Figure 3:**
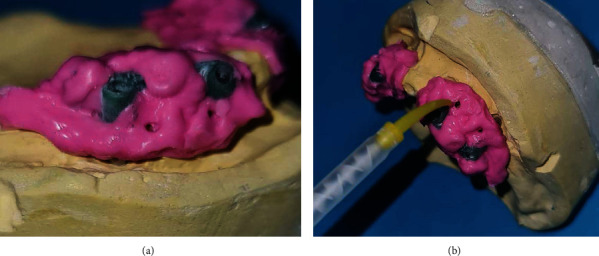
Laboratory process. (a) Resin pattern with the impression is connected to the analogs in the cast by screws. (b) Gingival mask is injected from the buccal holes.

**Figure 4:**
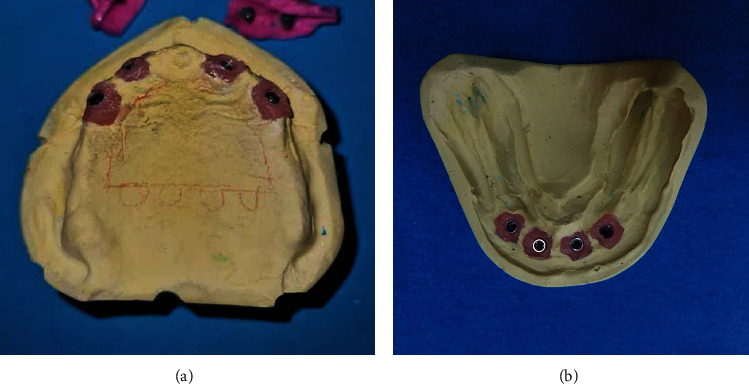
New gingival mask. (a) Maxillary cast. (b) Mandibular cast.

**Figure 5:**
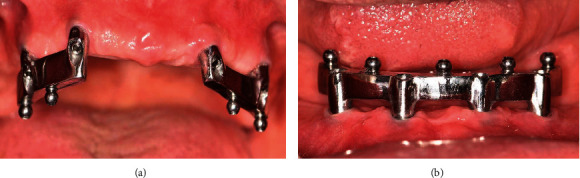
CAD/CAM bar and ball attachment. (a) Maxillary arch. (b) Mandibular arch.

1. Screw-retained resin pattern was tightened intraorally with 10 N/cm torque (Figure 1)
2. Light-body addition silicone impression material (Betasil Vario Light, Muller-Omicron GmbH & Co.KG, Lindlar, Germany) was injected around the resin pattern to approximately 1 cm distance
3. After setting of impression material, the resin pattern screws were untightened and the resin pattern along with the attached impression material was removed from the oral cavity (Figure 2)
4. One layer of separator (petroleum jelly) was applied on the internal surface of the impression material, and the resin pattern was screwed onto the cast with 10 N/cm torque
5. A round diamond bur was used to create one hole in the buccal and one hole in the lingual surface of the impression material of gingival mask. The hole in the buccal surface was used to inject the automix injectable gingival mask (GI Mask, Coltene/Whaledent Inc., Altstatten, Switzerland), and the hole in the lingual surface was used to allow air leakage and prevent air retention at the site (Figure 3). The injection was continued until the material leaked out through the lingual hole
6. After setting of gingival mask, the resin pattern along with the attached impression material was removed from the cast (Figure 4)
7. The rest of the procedure for the fabrication of milled bar and ball attachment was continued for the patient (Figure 5). The attachment was tightened intraorally on the implant, and proper relationship of abutments with surrounding soft tissue was clinically evaluated. Also in order to access for hygiene, the sufficient space between the bar attachment and the soft tissue was ensured

## 3. Discussion

Temporary prosthesis in completely edentulous patients is a diagnostic tool that provides acceptable function and aesthetics for patients until the final prosthesis is ready. They can be changed many times until the prosthesis is satisfactory for the patient. When a satisfactory result is obtained, the final prostheses should be copied of the temporary prostheses [17]. In a number of articles, to determine the emergence profile in fixed restoration, combined methods that include the use of temporary restoration and scanning are used [18–20]. It should be noted that the use of these methods is complicated in edentulous patient due to the difficulty of scanning the edentulous jaw and the absence of temporary restoration in overdentures. The advantages of the proposed technique include enabling refabrication of gingival mask in a short time and without requiring additional treatment sessions, or requiring reimpressions from the entire arch. The shortcomings of this technique include the possibility of void formation or incomplete recording of peri-implant tissue by the light-body impression material probably due to the lack of support of the light-body impression material. This problem occurred in the mandibular impression of the patient mentioned, which may lead to the construction of a superstructure with insufficient gingival compatibility. This problem is more important in patients with a fixed restoration treatment plan because it is more difficult to follow hygiene. To overcome this problem, it is recommended to use two-phase one-stage (putty+wash) impression technique (light-body impression material is injected at the site, and high-consistency putty impression material is packed over it). This technique can be used for both fixed and removable implant-supported restorations. It does not necessarily require a resin pattern, and an impression jig can be used instead. In the follow-up of this patient for one year, no signs of inflammation or peri-implantitis were found. In order to determine the accuracy of this method in a measurable way, it is suggested to compare the scan of the gingival mask obtained from this method with the scan of the original gingival mask in the future study.

## 4. Conclusion

Gingival mask plays an important role in the fabrication of an optimal restoration. The aim of this study was to provide a simple and low-cost solution to reconstruct the missing gingival mask without repeating the previous steps. This technique has two main stages. In the first stage, the resin pattern or impression jig is closed on the implants in the patient's mouth, and the impression material is injected around them. In the second stage, the resin pattern and impression material are closed on the cast and the gingival mask is injected. This technique has the following advantages:

- Using the previous cast of the patient and not requiring repetition of previous steps
- Saving time and cost for refabrication of gingival mask
- Applicability in both fixed and removable implant-supported restorations

The technique was effective in our patient and solved the problem, but due to the lack of articles in this field, it is suggested to conduct more studies with longer follow-up time.

## Figures and Tables

**Table 1:** Definition of abbreviations and techniques.

Abbreviations and techniques	Definition
CAD/CAM	Computer-aided design (CAD) and computer-aided manufacturing (CAM).
Suprastructure	All parts that are attached to an implant as a dental prosthesis.
Lost-wax technique	An ancient technique for making a precise replica of an object by casting it in molten metal.
Pickup technique	In this technique, the transfers remain encased in the impression material after the impression has been removed from the patient's mouth.

## Data Availability

The data used to support the findings of this study were supplied by Somayeh Zeighami under license and data will be available on request. Requests for access to these data should be made to Somayeh Zeighami.
